# Supplementary figures and images for: Rare heterozygous missense variants in VSX2 are associated with retinal detachment
Source: PLoS Genet. 2026 Feb 3;22(2):e1012027. doi: 10.1371/journal.pgen.1012027 (PMC12890223; doi:10.1371/journal.pgen.1012027)

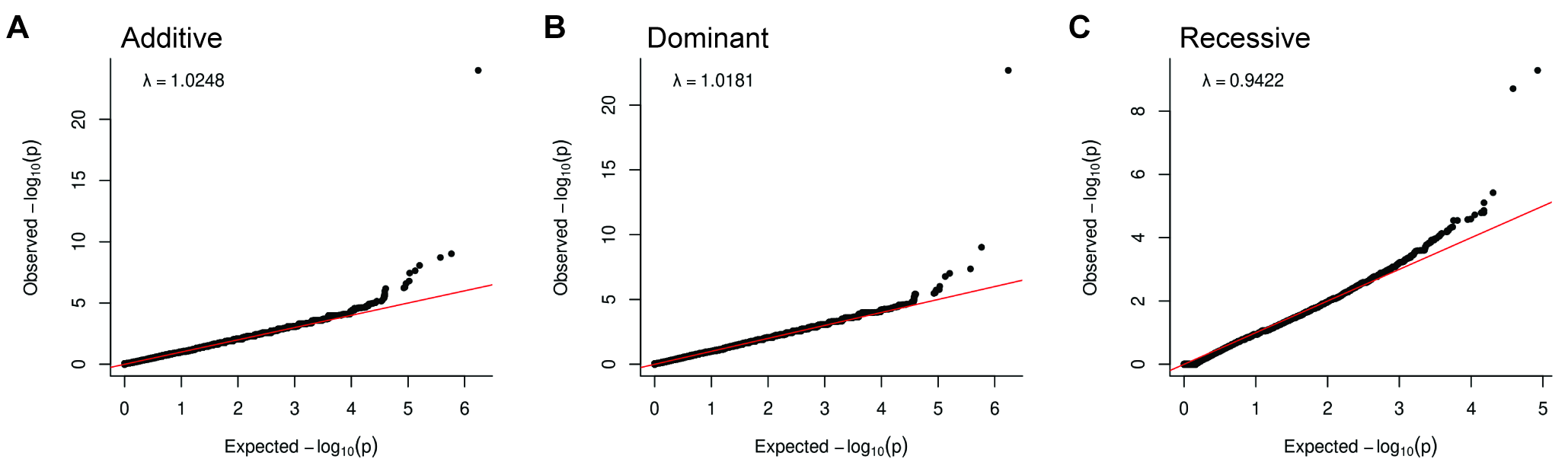

Supplement: S1 Fig — (A-C) Observed versus expected -log10(p) values for the additive, dominant, and recessive genetic models. The null-distribution of expected p-values is based on an n-of-1 permutation of case-control labels. (TIF) [file pgen.1012027.s001.tif]

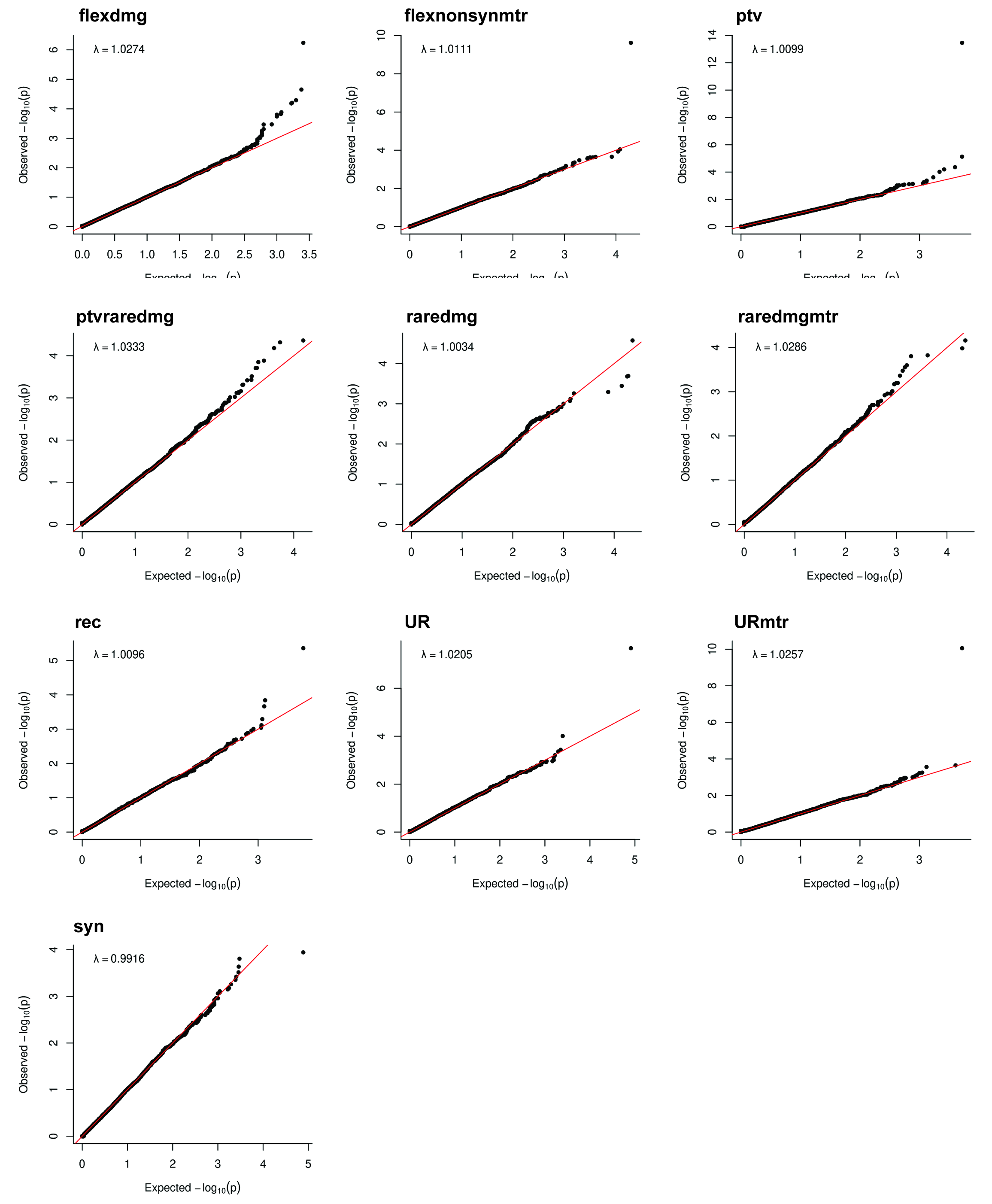

Supplement: S2 Fig — Observed versus expected -log10(p) values for all 11 collapsing analysis models. The null-distribution of expected p-values is based on an n-of-1 permutation of case-control labels. (TIF) [file pgen.1012027.s002.tif]

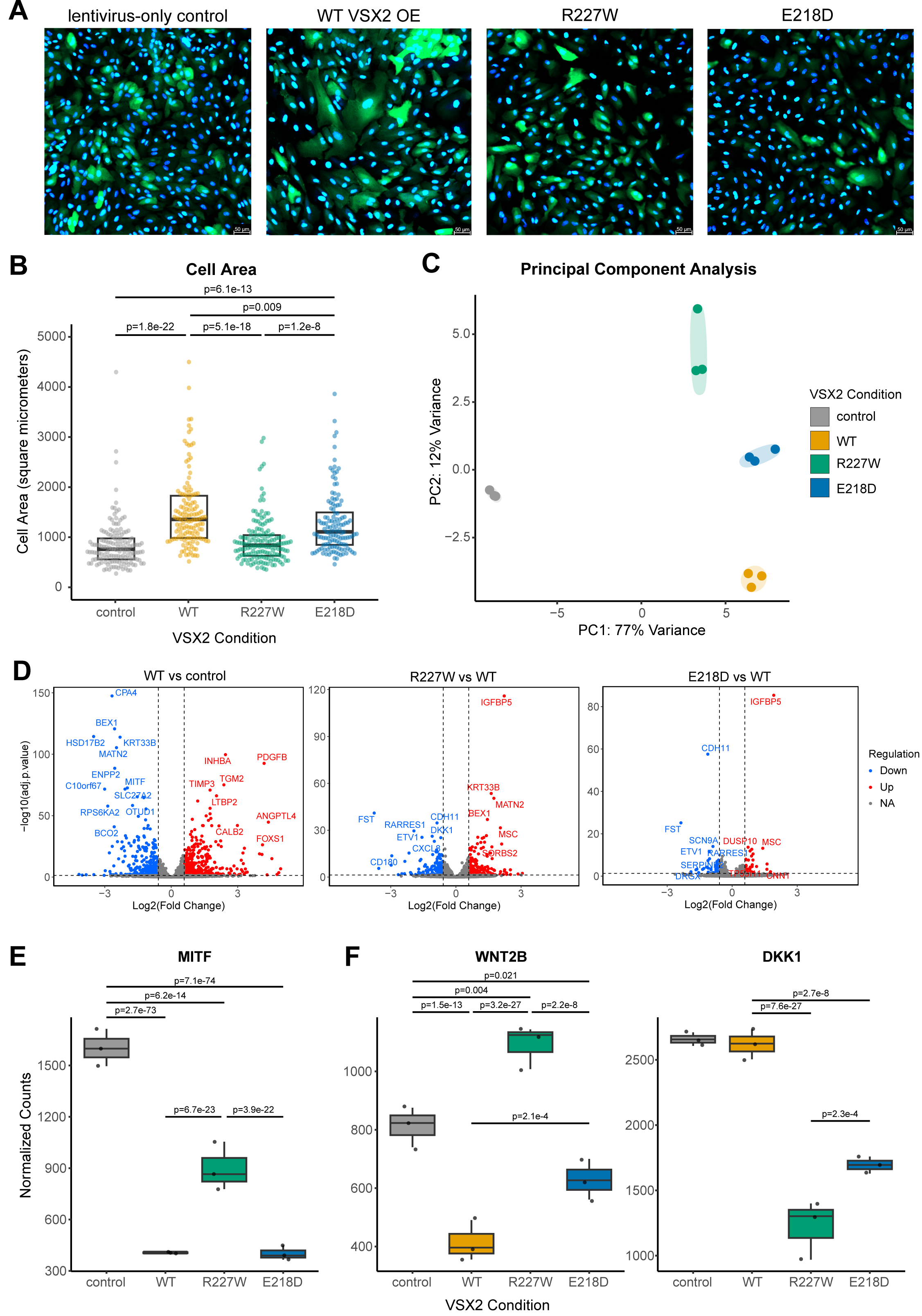

Supplement: S3 Fig — A) Representative fluorescent images of APRE-19 cells transduced with lentiviruses expressing WT or variant VSX2 constructs or a control vector (Blue = DAPI, green = eGFP). B) Quantification of cell area (µm 2) across experimental conditions. C) Principal component analysis of bulk RNA-seq data demonstrating distinct transcriptional clustering of APRE-19 cells expressing different VSX2 variants. D) Differential expression analysis comparing WT vs control, R227W vs WT, and E218D vs WT. E) Differential expression of MITF and F) WNT signaling genes across experimental conditions. (TIF) [file pgen.1012027.s003.tif]

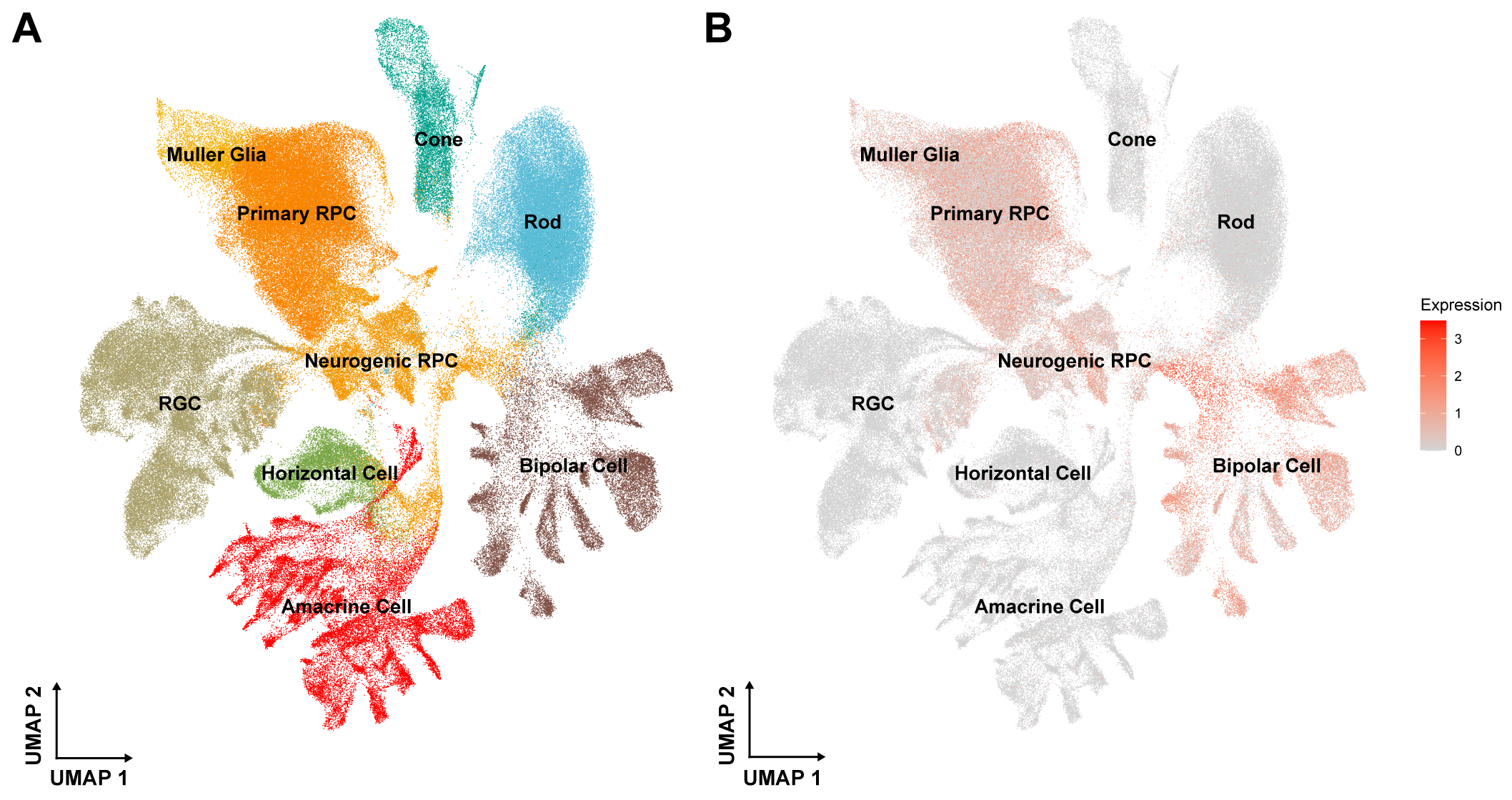

Supplement: S4 Fig — A) UMAP of developing human retinal cell types and B) UMAP highlighting VSX2 expression. (TIF) [file pgen.1012027.s004.tif]

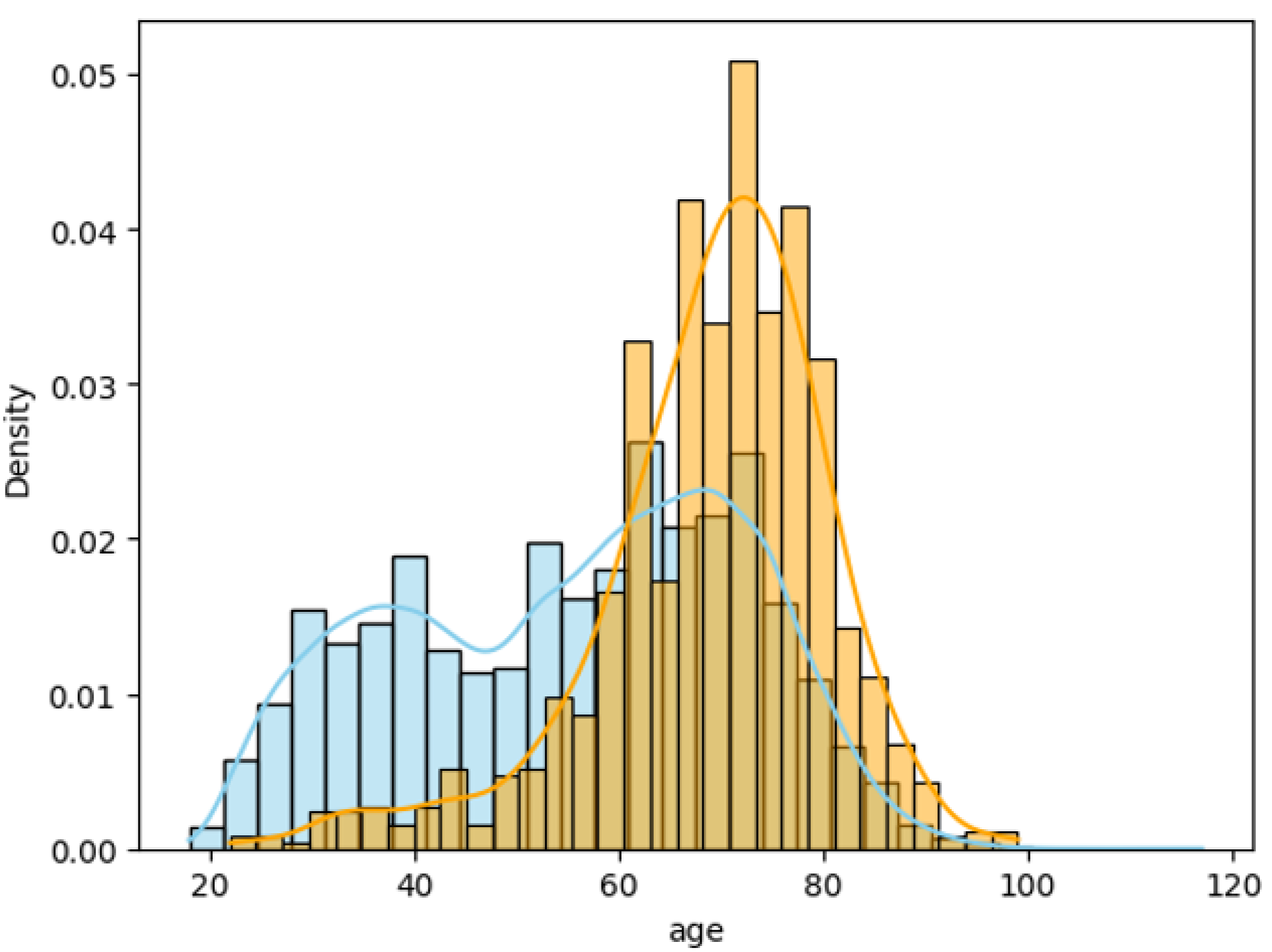

Supplement: S5 Fig — Note: this density plot was generated before age filtering and down-sampling in the All of Us Biobank. (TIF) [file pgen.1012027.s005.tif]
