# Supplementary material for: Rare heterozygous missense variants in VSX2 are associated with retinal detachment
Source: PLoS Genet. 2026 Feb 3;22(2):e1012027. doi: 10.1371/journal.pgen.1012027 (PMC12890223; doi:10.1371/journal.pgen.1012027)
Supplement: Attachment — Submitted filename: 2025-09-17_reviewer_response.docx [file pgen.1012027.s026.docx]

We thank the reviewers for their comments and appreciate their insightful feedback. Our responses to their comments are below. When referencing specific lines where new edits can be found, we are specifically referring to the line numbers in the unmarked Manuscript document (without track changes).

**Reviewer #1:**

This article discusses the results of a large-scale association study using data from the UK biobank regarding the potential role of VSX2 in retinal detachment. Overall, the study is well-designed, and the article is well written. I suggest a few minor edits.

1. A more detailed description of the genetic architecture of RD is warranted. While the authors quickly mention that in the intro, I believe a more detailed overview of what has been known so far would educate the readers better.

We have added the following to the introduction to elaborate further on the genetic architecture of RD (lines 93-102):

*“However, monogenic causes represent only a small minority of rhegmatogenous RD, likely accounting for ~1% of all cases^14^. Most RD cases arise from a combination of genetic variants and environmental or anatomical risk factors, like myopia^10^. Correlation analyses from prior RD GWAS support a shared genetic architecture between RD and its major epidemiologic risk factors, including high myopia and cataract operation^15^. Nonetheless, first-degree relatives of RD patients have approximately twice the lifetime risk of rhegmatogenous RD, independent of myopia^16^. Prior GWAS have identified numerous RD-associated loci, most of which are highly expressed in the neural retina or retinal pigmented epithelium^15,17^. Furthermore, many of the loci are broadly involved in extracellular matrix, cytoskeletal, and cell-adhesion pathways^18^.”*

2. Line 84: A reference is needed for evidence for retinal dystrophies and cataract (role of het variants)

We have added the citations Barton et al. 2022 and Heyne et al. 2023 for retinal dystrophies and cataract, respectively (line 114).

3. What other ICD10 codes related to eye disease were found in the RD cohort? What measures were taken to make sure no confounding phenotypes were present in those patients (did any have cataracts, glaucoma, retinal degeneration, etc.).

We have included a new supplementary table (Table S6) that provides the proportion of co-occurring ICD10 codes among RD cases compared to the entire cohort. In our original manuscript, we performed a conditional analysis to ensure the VSX2 association with RD was independent of myopia. In response to this reviewer’s comment, as well as a comment from Reviewer 2, we have extended this analysis to condition on cataract and glaucoma. Encouragingly, the VSX2 association with RD remained significant even after excluding individuals with co-morbid glaucoma and individuals who had been diagnosed with cataract prior to RD. We have included a summary of the statistics below, and include a full table of summary statistics as supplementary table 8 in the revised manuscript.

| **Analysis (model)** | **Gene/Genotype** | **Cohort** | **P-value** | **Odds Ratio [95% CI]** |
| --- | --- | --- | --- | --- |
| Collapsing (flexnonsynmtr) | *VSX2* | Full cohort | 2.4e-10 | 2.8 [2.1, 3.7] |
| Collapsing (flexnonsynmtr) | *VSX2* | Excluding cases with history of prior cataract | 3.31e-08 | 2.8 [2.0, 3.8] |
| Collapsing (flexnonsynmtr) | *VSX2* | Excluding glaucoma cases | 3.4e-10 | 2.9 [2.1, 3.9] |
| ExWAS (dominant) | *VSX2*  (14-74259676-G-T) | Full cohort | 1.8e-5 | 6.6 [2.9, 12.9] |
| ExWAS (dominant) | *VSX2*  (14-74259676-G-T) | Excluding cases with history of prior cataract | 7.6e-08 | 5.9 [3.2, 9.6] |
| ExWAS (dominant) | *VSX2*  (14-74259676-G-T) | Excluding glaucoma cases | 1.4e-08 | 5.8 [3.3, 9.6] |

4. Some abbreviations have not been described e.g. RPRS. Please make sure all abbreviations are detailed the first time they are mentioned.

We have revised the main text to ensure all abbreviations are detailed at the first instance.

**Reviewer #2:**

Retinal detachment (hereafter “RD”) is a potentially blinding condition that arises as a result of a widening gap between the neuroretina and the retinal pigmented epithelium. Prompt intervention may stabilize the condition, but not reverse any of its consequences that should medical attention be delayed, could be severe.

Statistical association between disease and genes are very useful not only because at a certain stage they may enable the prediction of individuals who are at higher disease risk and on whom medical attention can be prioritized, but they also inform about the underlying pathophysiological mechanisms underlying that condition, creating the premises for newer interventions and therapies.

While most genetic associations have focused on common variants (the so called “common variant – common disease” model), this approach tends to omits some of the strongest clinical effects and is generally inversely correlated with allele frequency, because rare variant characterization is limited by cost and technological availability. Increasingly population-based cohorts are offering opportunities for rare variant analyses, and these opportunities have been picked up by Brock and co-authors, who analyzed available exome sequencing information from the UK Biobank and two smaller (but in absolute terms large) cohorts.

The authors find association between rare coding variants and RD, and in addition to known genes implicated in RD, such as FAT3, RDH5 and COL2A1. They for the first time report an association with a missense variant within the coding sequence VSX2. Evidence that other missense variants within that gene may contribute at various degrees to RD is also reported. The authors conclude that these associations are proper to RD and not confounded from associations with other phenotypes.

This is a detailed work where the reader will find so much to admire. There are however question marks and potential limitations on which this review will be concentrating, in the hope of improving the clarity and potentially the impact of the report.

The main question that remains after reading the manuscript is whether the VSX2 Glu218Asp mutation is a primary RD gene or does so through its putative action through other channels. Results by Boutin et al. have found a very strong genetic correlation between RD on one side and both refractive error (r=0.25) and a cataract (r=0.26) on the other. This means that simple genetic risk scores calculated for any these conditions would be associated with RD – but whether they inform about RD pathogenesis may be more debatable. Variants within VSX2 (previously known as CHX10) have been previously linked to a syndromic combination of microphthalmia, cataract and iris colobomas. The variant identified in the originally reported families codes for the same protein domain, just 18 aminoacids away. It is possible therefore that VSX2 may cause RD indirectly, through its effects on refractive error AND cataract. The authors have explored one of these routes (myopia) but are not reporting any association with history of cataract operations. Stratifying for history of cataract operation would have been of great interest for the reader.

We appreciate the reviewer’s positive comments. In response to these helpful comments, we have now provided additional validations to demonstrate that the association between *VSX2* and RD is not secondary to cataract.

First, per the reviewer’s suggestion, we re-ran the VSX2 association test after excluding individuals who had a history of documented history of cataract surgery (n=9,553 total individuals). *VSX2* remained robustly associated with RD in both the collapsing analysis (OR=2.7, p=8.95e-9) and the ExWAS (OR=4.93, p=1.09e-7).

However, the surgical data in the UK Biobank is sparse, as it mostly relies on self-reported data at the time of enrollment and limited hospital billing code data. This is evident in the relatively small number of cases with a documented history of cataract surgery (n=10,000 out of ~450,000 participants,). We thus provided an additional, more conservative analysis in which we tested whether the association between *VSX2* and RD remained significant after excluding cases who had a documented history of cataract prior to being diagnosed with an RD. There were a total of 68,986 indiviudals with a documented date of diagnosis for cataract; this approach thus encompasses a larger, more diverse set of cataract cases. Encouragingly, the point estimate remained nearly identical to the original analysis, and the p-value remained significant despite the reduction in power:

| **Analysis (model)** | **Gene/Genotype** | **Cohort** | **P-value** | **Odds Ratio [95% CI]** |
| --- | --- | --- | --- | --- |
| Collapsing (flexnonsynmtr) | *VSX2* | Full cohort | 2.4e-10 | 2.8 [2.1, 3.7] |
| Collapsing (flexnonsynmtr) | *VSX2* | RD preceding cataract | 1.5e-6 | 3.2 [2.0, 4.8] |
| ExWAS (dominant) | *VSX2*  (14-74259676-G-T) | Full cohort | 1.8e-5 | 6.6 [2.9, 12.9] |
| ExWAS (dominant) | *VSX2*  (14-74259676-G-T) | RD preceding cataract | 9.3e-10 | 5.9 [3.4, 9.5] |

Thus, this analysis provides further evidence that the association between *VSX2* and RD appears to be independent of other known RD risk factors. Collectively, given that VSX2 variation appears to associate with RD independent of myopia and cataract risk, we believe that this association will provide key insight into RD pathogenesis.

Another question pertains to certain statistical analyses choices made by the authors. Controlling for potential confounders is very important, but this is done in ways which may be unusual and occasionally inconsistent. For example, the authors correctly used Mantel-Hanetzel stratified analyses, but they sex-match cases and controls, without analyzing them separately. They do not control for age, a well known risk factor, and rather selectively don’t report the age distribution among controls. It is unclear why slightly more tortuous solutions were found for situations in which many statisticians would have either stratified analyses, or linear / mixed model analyses, also adjusting for age.

Improving clarity on this issue is important for at least two reasons. First, the risk of ascertainment bias in cases and controls, and second, the absence of any description of the matching procedures could lead some readers to doubt whether there is any implicit risk of selection bias towards particular age-groups.

We appreciate the reviewer’s thoughtful comments regarding our statistical choices. Our analyses follow the rare variant framework that we first introduced in our UK Biobank rare variant study in *Nature* (Wang, Dhindsa et al., 2021), which has subsequently been applied by multiple groups. In this framework, associations are evaluated using Fisher’s exact test or the Cochran–Mantel–Haenszel (CMH) test, which we demonstrated to be more robust for rare variant analyses than traditional regression approaches in this setting.

Potential confounding was addressed through careful case–control harmonization. Specifically, we account for sex imbalance between cases and controls, since differential male–female ratios can introduce artifacts when evaluating sex chromosome variants. Matching on sex has been shown to mitigate this concern (Petrovski et al., *Am. J. Respir. Crit. Care Med.*, 2017). We also performed ancestry-stratified analyses to control for population stratification. The genomic inflation factor for our binary analyses was λGC = 1.02 (line 192), consistent with minimal systematic inflation.

Regarding age, the median age at enrolment was 60 years among cases versus 56 years among controls. Although some younger controls may develop retinal detachment in the future, inclusion of such individuals would be expected to attenuate rather than inflate our observed effect sizes. To further evaluate robustness, we performed an additional analysis using Firth logistic regression including age and sex as covariates. The effect estimates were nearly unchanged, supporting the age-independence of the association.

| **Model** | **P-value** | **Odds ratio** |
| --- | --- | --- |
| Collapsing (flexnonsynmtr) | 1.6e-10 | 2.8 [2.1, 3.7] |
| ExWAS | 4.6e-10 | 6.1 [3.7, 9.5] |

The authors report the results of an analysis of “overrepresentation” and “enrichment” of variants linked to other ocular conditions with RD. Some readers may not agree with the application of these labels to the analyses conducted. An argument can be made that these are simple pleiotropy tests and a formal statistical test for enrichment or more-than-expected occurrence of these statistical associations is not reported. It is reasonable to expect some overrepresentation due to the multifactorial nature of RD etiology, but evidence presented is not unambiguously clear.

In our revision, we clarified the statistical objective and analysis used. Our goal was to assess whether Mendelian ocular disease genes were represented among our nominally significant RD-associated genes more frequently than expected by chance. To address this formally, we applied Fisher’s exact test comparing the observed overlap with the null expectation. We have revised the Results text to state explicitly that this constitutes a formal overrepresentation test (lines 409-11), rather than implying enrichment indirectly. This clarification is intended to make the statistical framework and terminology unambiguous to the reader.

*“After excluding Stickler syndrome-associated genes, which have well-established associations with RD, we tested whether this overlap exceeded random expectation using Fisher’s exact test (Methods).”*

Less important instances where more clarity would be beneficial are below. The authors report checking concordance with previous genotypes released for the same individuals. It may be better to explain the logic behind this, but also the choice of the thresholds: 0.49 and 0.465, it is difficult to understand why such choices were made. Again, an argument could have been made to remove all samples that were not 99% concordant with previous genotyping versions of themselves, but it is possible there is a problem of clarity and the way the thoughts were phrased.

To improve clarity, we now emphasize that these thresholds were not arbitrary but were adopted directly from our previously published quality-control pipelines introduced in Wang, Dhindsa, et *al.* (*Nature*, 2021), where they were empirically calculated using UK Biobank WGS, exome, and array data. These kinship-based cutoffs account for expected variation in call sets across platforms while identifying mismatched or swapped samples. Note that KING kinship coefficients are scaled such that duplicate samples have kinship 0.5, not 1. Thus our chosen thresholds are in-line with the reviewer’s suggestion that we remove samples that are not roughly >99% concordant.

We have updated the methods to reflect this rationale in the (lines 633-36):

*“KING kinship coefficients are scaled such that duplicate samples have kinship 0.5.* *We thus removed samples with kinship <0.49 to exome data or <0.465 to array data – empirically derived thresholds that account for platform-specific variation and reliably identify mismatches and sample swaps^89^.”*

The authors write that “there were significant (p<1x10-8) associations” and that the genomic inflation factors were reasonably tame. Both these notions are linked in different ways to expectations about linkage disequilibrium in a population. While there is clarity of what the level of significance we should expect for common variants, it would be useful to have a description of how the authors picked that level of significance for their study.

We thank the reviewer for raising this point. To clarify, we implemented the significance threshold p<1x10-8 based on our prior 2022 study, in which we empirically determined significance thresholds for rare-variant collapsing analyses in the UK Biobank using two approaches. We first evaluated the p-value distribution of a synonymous collapsing model across 18,762 genes and >300 million tests to serve as an empirical null. The extreme tail of this null distribution did not extend beyond ~2.2×10⁻⁸ except for a small number of biologically plausible associations. We also performed >3 billion of n-of-1 permuted collapsing tests across all models. The minimum permutation-based p-values were 1.9×10⁻⁹ for binary traits and 3.2×10⁻⁹ for quantitative traits. Because p-values below these thresholds did not occur under the null in either approach-even when accounting for linkage disequilibrium and correlation among models, we adopted p < 1×10⁻⁸ as a conservative study-wide threshold for downstream collapsing analyses.

The authors write that “a stronger association with serous RD (p=5.6x10-5; OR [95% CI] = 3.4 [1.9-5.8]) than with rhegmatogenous RD (p=2.7x10-4; OR [95% CI] = 2.6 [1.5-4.1])”. Yet the confidence intervals largely overlap.

We have now revised the text as follows (lines 275-278):

*“Under the flexnonsynmtr model, we observed an association of VSX2 with both serous RD (p=5.6x10^-5^; OR [95% CI] = 3.4 [1.9-5.8]) and rhegmatogenous RD (p=2.7x10^-4^; OR [95% CI] = 2.6 [1.5-4.1]), with a higher OR for serous RD, though the confidence intervals largely overlap (Fig 3B).”*

Tables and figures legends may be improved. For example "FF, FT and TT, OddsUCI" in one of the tables are not acronyms the reader would immediately relate to.

We appreciate the reviewer’s suggestion to improve the clarity of our table and figure legends. To address this, we have added explicit abbreviation definitions to the supplemental table legends to clearly explain all non-standard terms (e.g., FF, FT, TF, TT denote the four cells of the 2x2 contingency table from fisher’s exact test, and oddsLCI and oddsUCI indicate the lower and upper bounds of the 95% confidence interval for the odds ratio, respectively).

**Reviewer #3:**

Brock and Dhindsa et al. details a genome-wide sequencing study on retinal detachment, using 7,000 cases from the UK Biobank and replication cohorts. The authors show rare heterozygous missense variants in VSX2 as a risk factor for RD, a gene previously associated with recessive developmental eye disorders.

Major Comments:
Rare variants typically confer larger effect sizes and can provide clear mechanism and
clinical impacts than on common variants. It would be helpful to review the following manuscript in light of this work:
• Lupski, J. R., Belmont, J. W., Boerwinkle, E. & Gibbs, R. A. Clan genomics and the complex architecture of human disease. Cell 147, 32–43 (2011).

We thank the reviewer for this suggestion and agree that the framework outlined in the above paper provides important conceptual context for understanding the contribution of rare variants to disease. In the revised manuscript, we now cite this important work in our discussion of rare variants in the introduction (line 105).

The gene dosage model for VSX2 is well documented and discussed in the manuscript. It would be helpful to more explicitly connect these findings to other heterozygous variants in recessive disease genes manifesting as milder, or late-onset phenotypes. Possibly detail these other examples in the manuscript in the discussion.

We thank the reviewer for this suggestion. Although we included examples such as *LTBP2,* *LOXL3*, and P3H2 in the Results (lines 414-417), we agree that these findings warrant further discussion and connection to the *VSX2* gene dosage model we proposed. Thus, we have added the following to the revised Discussion (lines 524-529):

*“Furthermore, for several other recessive ocular disease genes such as LTBP2, LOXL3, and P3H2, our findings support a broader pattern in ocular genetics in which heterozygous variants contribute to milder or later-onset phenotypes like RD. Collectively, these observations reinforce a gene-dosage spectrum that likely extends beyond VSX2 and may have larger implications for interpreting genetic risk factors for complex eye diseases.”*

Were copy number variants or other structural variants looked for or studied? This may be helpful to find other cases with VSX2 variation.

In this study, we did not look at copy number variants or structural variants in this study, instead focusing on SNVs. Applying collapsing analysis frameworks to CNVs is an area we are actively exploring, however. Structural variants in *VSX2* are likely under detected in our study but represent a future important direction.

It is interesting that the effect size estimates (particularly for the p.Glu218Asp variant) differ so much between the different cohorts. It would be useful to discuss the potential reasons for this difference (sequencing depth, phenotypic delineation differences, etc?)

We thank the reviewer for this helpful suggestion and have added the following brief phrase to the Results to highlight another possible source of variation in addition to differences in cohort sizes (lines 229 - 232):

*“Despite their smaller sample sizes and reduced statistical power relative to UKB, as well as differences in phenotypic ascertainment, both cohorts yielded effects that were directionally concordant with the UKB association (100kGP: OR = 15.65, p = 0.011; AoU: OR = 5.2, p = 0.067) (****S5 Table****).”*

Besides the notable differences in sample sizes across the cohorts, differences in phenotyping are also a likely contributor to the effect size estimate differences. For example, UK Biobank, All of Us, and 100kGP rely on distinct combinations of ICD coding practices, clinical adjudication, self-report surveys, and availability of procedure codes or imaging-confirmed diagnoses. These differences in diagnosis and reporting can lead to variation in case definition, which likely influences observed effect sizes for rare variants such as p.Glu218Asp in *VSX2*.

Additionally, have you thought about any functional validation especially for the p.Glu218Asp variant?

We thank the reviewer for this constructive suggestion. As requested, we performed functional assays by overexpressing wild-type (WT) and mutant *VSX2* constructs in the ARPE-19 retinal pigment epithelial cell line. We selected this model because it lacks endogenous *VSX2* but expresses established targets such as *MITF*, providing a clean background to assess transcriptional activity.

These new experiments are presented in the Results (lines 336-356), Discussion (lines 479-498), and Supplementary Figure 3.

Summary of Findings:

1. Validation of System: WT *VSX2* functioned as expected, strongly repressing *MITF* and regulating WNT pathway components (downregulating *WNT2B*, maintaining *DKK1*).
2. Differentiation from Severe Alleles: The microphthalmia-associated R227W variant (known to have impaired DNA binding) failed to repress *MITF* or regulate WNT targets, confirming its loss-of-function status.
3. Specific Defect of E218D: Crucially, the E218D variant displayed a selective functional impairment. It retained full repressive capacity against *MITF* (indistinguishable from WT) but exhibited significantly impaired regulation of WNT signaling and cell morphology.

Conclusion: These data suggest that E218D functions as a hypomorphic allele with an intermediate functional profile. The preservation of *MITF* repression may explain why E218D carriers do not exhibit microphthalmia (which is driven by *MITF* dysregulation), while the compromised WNT regulation offers a plausible mechanism for the observed retinal dysfunction and structural thinning.

**Supplemental Figure 3. Functional assessment of *VSX2* variants in APRE-19 cells.** **A)** Representative fluorescent images of APRE-19 cells transduced with lentiviruses expressing WT or variant *VSX2* constructs or a control vector (Blue = DAPI, green = eGFP). **B)** Quantification of cell area (µm^2^) across experimental conditions. **C)** Principal component analysis of bulk RNA-seq data demonstrating distinct transcriptional clustering of APRE-19 cells expressing different *VSX2* variants. **D)** Differential expression analysis comparing WT vs control, R227W vs WT, and E218D vs WT. **E)** Differential expression of *MITF* and **F)** WNT signaling genes across experimental conditions.

Have you looked through ClinVar or other clinical variant databases to see how VSX2 is represented?
• Landrum, M. J. et al. ClinVar: improving access to variant interpretations and supporting evidence. Nucleic Acids Res. 46, D1062–D1067 (2018).

Yes, we examined ClinVar, which contains multiple pathogenic and likely pathogenic variants. All previously-documented pathogenic variants from ClinVar and clinical case reports are visualized in the lollipop plot in Figure 4B. However, almost all variants are associated with severe developmental eye phenotypes such as microphthalmia and/or coloboma. We did not identify any ClinVar submissions associating *VSX2* with retinal detachment.
